# Prescription patterns of comedications associated with drug-drug interactions risk in HCV-infected patients undergoing direct-acting antiviral treatment: an analysis of an administrative claims database in Japan

**DOI:** 10.1186/s40780-025-00442-5

**Published:** 2025-04-18

**Authors:** Daisuke Nakamoto, Yi Piao, Hajime Mizutani, Ryozo Wakabayashi, Satoshi Otokita, Alice Stead, Candido Hernandez, Masahisa Jinushi

**Affiliations:** 1 Gilead Sciences K.K., 1-9-2 Marunochi Gran Tokyo South Tower 16F, Chiyoda-ku, Tokyo, 100-6616 Japan; 2 Datack, Inc, 707, 1-8-9, Iidabashi Chiyoda-ku, Tokyo, 102-0072 Japan; 3 Gilead Sciences, Inc, 333 Lakeside Drive, Foster City, CA 94404 USA

**Keywords:** Hepatitis C virus, Direct-acting antiviral agents, Drug-drug interactions, Sofosbuvir/velpatasvir, Glecaprevir/pibrentasvir

## Abstract

**Introduction:**

While direct-acting antivirals (DAA) are effective treatment for hepatitis C virus (HCV) patients, concerns about drug-drug interactions (DDIs) remain a significant challenge. Although there are several studies investigating the risk of DDIs associated with DAA therapy, there is limited research evaluating DDIs of DAA therapy in real-world settings in Japan. We investigated prescription patterns of comedication associated with DDIs risk in HCV patients receiving DAA therapy using a large Japanese database.

**Methods:**

This was a descriptive epidemiological study, using the Japanese administrative claims database provided by DeSC Healthcare, Inc. Patients who initiated sofosbuvir/velpatasvir (SOF/VEL) or glecaprevir/pibrentasvir (GLE/PIB) between April 2017 and August 2023 were identified from the data. The primary outcome was DDIs associated with comedications which were assessed based on both Japanese package inserts and the Liverpool HEP Drug Interaction Checker (Liverpool HEP checker).

**Results:**

Patients included in this study were 7,338, with 467 prescribed SOF/VEL and 6,871 prescribed GLE/PIB. The mean age of the patients was 69.9 years (SD = 13.1), with 50% being male. The median number of comedications was higher in the SOF/VEL group (14.0; IQR = 14.0) than in the GLE/PIB group (9.0; IQR = 12.0) and based on package insert and Liverpool HEP checker, the DDI risk was present in 59.3% (277) of the SOF/VEL group and 51.5% (3,542) of the GLE/PIB group. DDI risk involving two or more medications in combination with a DAA was 14.1% (66) in the SOF/VEL group and 24.0% (1,648) in the GLE/PIB group. In terms of DDI severity, in the SOF/VEL group there were no patients identified under the level “Contraindication (Red)” category, indicating medications that do not co-administered, in contrast with the 1.7% (115) in the GLE/PIB group who were identified as “contraindication (red)”.

**Conclusion:**

A considerable proportion of patients were prescribed medications with DDI risk during DAA treatment. A small but notable proportion of patients were on “Contraindication (Red)” medications. Consideration of the potential DDI risks associated with comedications by healthcare professionals is advised, referring not only to package inserts but also tools such as Liverpool HEP checker to guide safe prescribing when initiating DAA therapy for HCV patients.

**Supplementary Information:**

The online version contains supplementary material available at 10.1186/s40780-025-00442-5.

## Introduction

Direct-acting antiviral (DAA) agents have significantly improved the treatment of hepatitis C virus (HCV), allowing for high safety and sustained virologic response (SVR) rates. Currently, the two IFN-free DAA therapies mainly used in Japan are glecaprevir/pibrentasvir (GLE/PIB) and sofosbuvir/velpatasvir (SOF/VEL) [1]. Despite the availability of innovative treatments such as GLE/PIB and SOF/VEL, an estimated 304,220 to 629,437 untreated HCV patients are expected to remain by 2025 [2, 3].

While DAAs are an effective treatment for HCV patients, concerns about drug-drug interactions (DDIs) remain a significant challenge. The aging HCV population in Japan, with increasing comorbidities and concomitant medication use, underscores the growing importance of disease management strategies [4]. DDIs are known to be a major cause of adverse events, in addition to potentially reducing or increasing the therapeutic effects of drugs [5]. It has been reported that DDIs are responsible for 2–18% of adverse events [6, 7, 8, 9] and 54% of these events are preventable [8]. While DDIs can be avoided in HCV treatment, it is essential for healthcare providers (HCPs) to understand the comorbidities and comedication profiles of HCV patients to optimally manage HCV infection in line with patient-specific characteristics [10]. Thetreatment guidelines from the American Association for the Study of Liver Diseases (AASLD) and the European Association for the Study of the Liver (EASL) recommend a thorough review of medications in all HCV patients to assess the DDI risk before starting DAA therapy or any other medications during treatment [11, 12]. For a comprehensive assessment, the use of Liverpool University HEP Drug Interaction Checker tool (Liverpool HEP Checker), which allows the assessment of DDIs for over 900 drugs is recommended by EASL [13]. On the other hand, the safety profile of DAAs listed in Japanese package inserts and Japan Society of Hepatology (JSH) guidelines covers a limited range of approximately 40 drugs studied in clinical trials [1], and the importance of more detailed DDI assessments in clinical practice is suggested [4].

Several studies have investigated the risk of DDIs associated with DAA therapy in patients with HCV [14, 15, 16, 17, 18, 19, 20, 21]. For example, an observational study from Italy [14] and another from Spain [15] showed that a lower proportion of SOF/VEL patients had concomitant medications with DDI risk compared to GLE/PIB patients. Conversely, another Italian study [16] reported a slightly higher proportion of SOF/VEL patients with DDI risk, although a greater number of GLE/PIB patients had contraindicated medications. Despite these prior studies, there is limited research evaluating DDIs in real-world settings of DAA therapy in Japan. Although one real-world study exists [4], it used data collected before the introduction of GLE/PIB and SOF/VEL and was limited to institutions where patients received DAA treatment. Therefore, a comprehensive evaluation, including data from facilities outside DAA treatment centers, has not yet been conducted. The HCV patient population in Japan is older and often presents with multiple comorbidities and polypharmacy [22], differing from Western countries where younger populations affected by the opioid epidemic are more common [23, 24]. Consequently, status of the DDI risk in Japan is expected to differ from that in Western countries. Japan may increase the possibility to achieve the World Health Organization (WHO)’s target of HCV elimination by 2030. To support this goal, it is important to expand access to DAAs for patients without potential safety concerns.

In this study, we investigated the real-world prescription patterns of comedication associated with DDIs risk in HCV patients receiving DAA therapy using a large Japanese database, including elderly patients. We assessed DDIs based on both Japanese package inserts and the Liverpool HEP Checker.

## Methods

### Study design and data source

**Fig. 1:**
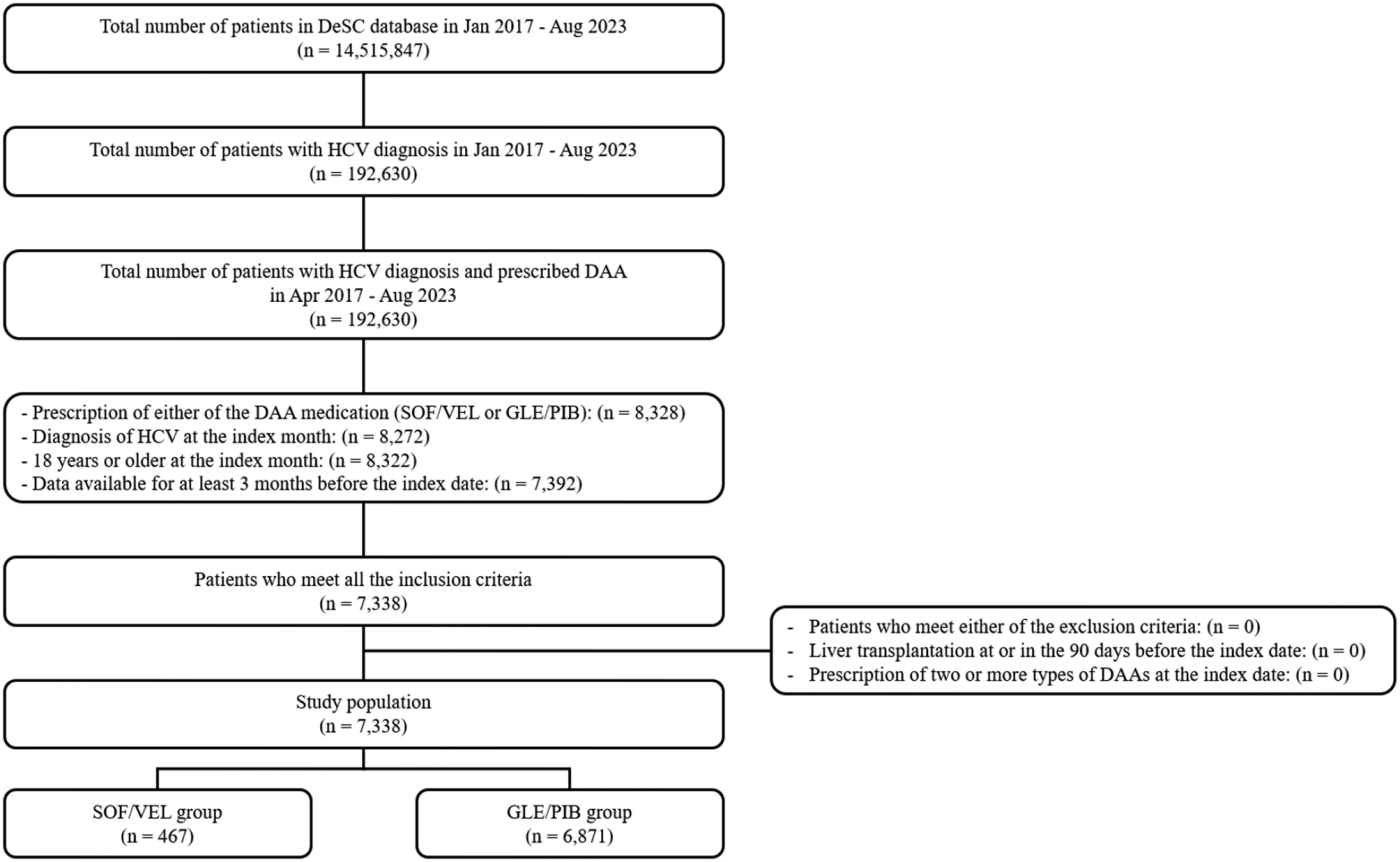
Patient selection flow

This is a descriptive epidemiological study, using the Japanese administrative claims database provided by DeSC Healthcare, Inc.Tokyo, Japan (DeSC). This database includes information on patients’ treatment and diagnosis, gathered from three types of insurers: Society-managed, employment-based health insurance association (SHI = Kenpo), National Health Insurance (NHI = Kokuho), and Latter-Stage Elderly Healthcare System (LSEHS = Koki Koreisha Iryo Seido). Therefore, the database comprises patients from diverse age groups and socioeconomic backgrounds. The database includes approximately 14.5 million individuals, as shown in Fig. 1. The study period was from January 2017 to August 2023. The identification period, defined as the timeframe for enrolling patients in the study, extended from April 2017 to August 2023. Note that this is because the DeSC database is a relatively new database with substantial data missing prior to 2017.

### Study population

The study population contained patients with HCV who initiated specific DAA therapy (SOF/VEL or GLE/PIB) in the identification period. The index date was the first prescription date for the DAAs. Patients were included if they met all of the following criteria: being prescribed a specific DAA therapy (SOF/VEL: WHO Anatomical Therapeutic Chemical (ATC) code J05AP55, GLE/PIB: WHO ATC code: J05AP57) during the identification period, being 18 years of age or older, having a diagnosis of HCV (ICD-10 code: B18.2) during the month of the index date, and having data available for at least three months prior to the index month. This approach stems from the Japanese healthcare system, which allows a maximum of three months of medication per prescription. Patients were excluded if they had a record of prescriptions of two or more types of DAA drugs at the index date or a record of liver transplantation (Living donor partial liver transplantation: K697-5, Liver collection for transplantation: K697-6, Allogeneic cadaveric liver transplantation: K697-7) at the index date or in the 90 days prior to the index date.

The study population was categorized into two exposure groups based on the type of DAA drugs at the index date: a SOF/VEL group and a GLE/PIB group. Follow-up for each patient started on the index date and ended when any of the following occurred: the discontinuation of DAA treatment, the prescription of a different DAA drug than the original one, the patient’s exit from the insurance, or end date of the study period. The duration of treatment was calculated by summing the “number of days’ supply” from all prescription records of DAA drugs in the continuous treatment. DAA therapy was considered continuous if the gap between the end date of the previous prescription (prescription date plus “number of days’ supply” minus one day) and the next prescription date is less than seven days. If this gap is equal or exceeds seven days, the treatment was considered as continuous only up to the end of the previous prescription.

### Outcomes

The primary outcome was DDIs associated with comedications. Comedications prescribed during the follow-up period, corresponding to the DAA treatment period, were evaluated. DDI was defined as the presence of any interaction listed in the Liverpool HEP Checker or as contraindicated or requiring caution according to the Japanese package insert. Potential DDIs were defined as any comedication not classified as a DDI in the Japanese package inserts but categorized as a DDI by the Liverpool HEP Checker. The severity of DDIs was also determined based on the categories of Liverpool University HEP Drug Interaction Checker as follows: Contraindication (Red), Potential clinically significant interaction (Amber), or Weak interaction (Yellow). If a drug was not listed in the Liverpool database, we classified it according to the Japanese package insert: “contraindication” was categorized as “Contraindication (Red)”, and “requires caution” was categorized as a “Potential clinically significant interaction (Amber)”. When multiple medications with varying severity levels were present, the most severe category was assigned.

We also collected information on age, gender, comorbidities, number of medications (in the 3 months prior to the index month), medical institution information (number of beds), severity of HCV (decompensated cirrhosis, compensated cirrhosis, chronic hepatitis C, others).

### Statistical analysis

A flow diagram was created to show the number of patients at each stage of the selection process. Descriptive statistics of patient characteristics were summarized, with means and standard deviations (SD) (or medians and interquartile ranges (IQR)) for continuous variables and frequencies and percentages for categorical variables. The number and proportion of cases corresponding to DDIs, potential DDIs, and the severity of DDIs were summarized in each treatment group. Additionally, the number and proportion of cases involving comedications classified as “Contraindication (Red)” were calculated. We also conducted an analysis of DDIs stratified by comorbidities and HCV severity. Data processing was executed using the Amazon Athena engine, version 3 (Amazon.com, Inc.). Statistical analyses were conducted using R, version 4.2.1 (The R Project for Statistical Computing).

#### Ethics

This research adhered to the ethical principles outlined in the World Medical Association’s Declaration of Helsinki, ensuring compliance with standards for studies involving human participants. Since the study used only de-identified data, there was no requirement to obtain patient consent or approval from an institutional review board or independent ethics committee, in accordance with the Japanese Ethical Guidelines for Medical and Health Research Involving Human Subjects.

## Results

Out of 14,515,847 patients in the DeSC database, 7,338 were included in this study, with 467 prescribed SOF/VEL and 6,871 prescribed GLE/PIB. Figure 1 illustrates the attrition process.

Table 1 presents the characteristics of patients. The overall mean age of the participants was 69.9 years (SD = 13.1), with 50% being male. Among the study population, the most commonly observed comorbidities were malignancy, peptic ulcer disease and congestive heart failure, affecting 1,612 patients (22.0%), 1,491 patients (20.3%), and 1,382 patients (18.8%) respectively. The median score on the Charlson Comorbidity Index (CCI) across all patients was 3.0 (IQR = 2.0). The median number of comedications (in the 3 months prior to the index month) was 9 (IQR = 11) The mean age was higher in the SOF/VEL group (73.8 years; SD = 10.5) than the GLE/PIB group (69.7 years; SD = 13.3). The SOF/VEL group had higher percentages of these conditions and median CCI score of 5.0 (IQR = 2.0) compared with 3.0 (IQR = 2.0) in the GLE/PIB group. The median number of comedications in the 3 months prior to the index month was higher in the SOF/VEL group (14.0; IQR = 14.0) than in the GLE/PIB group (9.0; IQR = 12.0).

**Table 1:** Characteristics of study population

	Overall		SOF/VEL		GLE/PIB	
Total	7,338		467		6,871	
Age (mean, sd)	69.9	(13.1)	73.8	(10.5)	69.7	(13.3)
Age categories, n(%)						
18–39	222	(3.0)	2	(0.4)	220	(3.2)
40–64	1,826	(24.9)	81	(17.3)	1,745	(25.4)
65–74	1,745	(23.8)	111	(23.8)	1,634	(23.8)
75 -	3,545	(48.3)	273	(58.5)	3,272	(47.6)
Sex, n(%)						
Male	3,671	(50.0)	215	(46.0)	3,456	(50.3)
Female	3,667	(50.0)	252	(54.0)	3,415	(49.7)
Observation period (median, interquartile range)	881	(885.8)	661.0	(921.5)	900.0	(887.0)
Comorbidities, n(%)						
Myocardial infarction	161	(2.2)	7	(1.5)	154	(2.2)
Congestive heart failure	1,382	(18.8)	111	(23.8)	1,271	(18.5)
Peripheral vascular disease	772	(10.5)	37	(7.9)	735	(10.7)
Cerebrovascular disease	1,079	(14.7)	62	(13.3)	1,017	(14.8)
Dementia	284	(3.9)	26	(5.6)	258	(3.8)
Chronic pulmonary disease	1,280	(17.4)	94	(20.1)	1,186	(17.3)
Rheumatic disease	296	(4.0)	18	(3.9)	278	(4.0)
Peptic ulcer disease	1,491	(20.3)	145	(31.0)	1,346	(19.6)
Mild liver disease	7,338	(100.0)	467	(100.0)	6,871	(100.0)
Diabetes without chronic complication	512	(7.0)	46	(9.9)	466	(6.8)
Diabetes with chronic complication	696	(9.5)	36	(7.7)	660	(9.6)
Hemiplegia or paraplegia	71	(1.0)	1	(0.2)	70	(1.0)
Renal disease	731	(10.0)	41	(8.8)	690	(10.0)
Any malignancy	1,612	(22.0)	190	(40.7)	1,422	(20.7)
Moderate or severe liver disease	492	(6.7)	254	(54.4)	238	(3.5)
Metastatic solid tumor	118	(1.6)	7	(1.5)	111	(1.6)
AIDS/HIV	18	(0.2)	1	(0.2)	17	(0.2)
Charlson comorbidity index (CCI) score (median, interquartile range)	3.0	(2.0)	5.0	(2.0)	3.0	(2.0)
Number of comedication (median, interquartile range)	9	(11.0)	14.0	(14.0)	9.0	(12.0)
Medical institution information: Number of the beds, n(%)						
unknown	29	(0.4)	0	(0)	29	(0.4)
< 20	772	(10.5)	30	(6.4)	742	(10.8)
20–99	200	(2.7)	8	(1.7)	192	(2.8)
100–199	724	(9.9)	36	(7.7)	688	(10.0)
200–299	586	(8.0)	27	(5.8)	559	(8.1)
300–399	1,100	(15.0)	54	(11.6)	1,046	(15.2)
400–500	784	(10.7)	51	(10.9)	733	(10.7)
500 <=	3,143	(42.8)	261	(55.9)	2,882	(41.9)
Medical institution information: Hub hospital for cancer treatment, n(%)						
Hub hospital for cancer treatment	3,327	(45.3)	273	(58.5)	3,054	(44.4)
Type of HCV, n(%)						
Decompensated cirrhosis	149	(2.0)	129	(27.6)	20	(0.3)
Compensated cirrhosis	234	(3.2)	10	(2.1)	224	(3.3)
Chronic hepatitis C	5,963	(81.3)	247	(52.9)	5,716	(83.2)
Others	992	(13.5)	81	(17.3)	911	(13.3)

Table 2 shows the DDIs during the follow-up period. DDIs were present in 277 patients (59.3%) of the SOF/VEL group and 3,542 patients (51.5%) of the GLE/PIB group during the follow-up period. DDIs involving two or more medications in combination with a DAA agent were 66 (14.1%) for the SOF/VEL group and 1,648 (24.0%) for the GLE/PIB group.

**Table 2:** DDIs associated with comedications

	Overall		SOF/VEL		GLE/PIB	
Total	7,338		467		6,871	
DDIs	3,819	52.0%	277	59.3%	3,542	51.5%
DDIs > = 2 medications	1,714	23.4%	66	14.1%	1,648	24.0%

The denominators for percentage calculation were the total number of cases in each group

Table 3 shows the potential DDIs during the follow-up period. The proportion of patients with potential DDIs (drugs not listed in the package insert but flagged by the HEP checker for DDI risk) was 14.6% in the SOF/VEL group and 28.5% in the GLE/PIB group.

**Table 3:** Potential DDIs associated with comedications

	Overall		SOF/VEL		GLE/PIB	
Total	7,338		467		6,871	
Potential DDIs	2,027	27.6%	68	14.6%	1,959	28.5%

The denominators for percentage calculation were the total number of cases in each group

Table 4 presents the observed numbers and proportions of patients at each severity level of DDIs associated with comedications during follow-up. ​In the SOF/VEL group, there were no patients who had identified as the highest severity level of “Contraindication (Red)”, and most (59.1%) were identified as the “Potential clinically significant interaction (Amber)”. In contrast, 115 patients (1.7%) in the GLE/PIB group were identified as “Contraindication (Red)” and 2061 patients (30.0%) were identified as “Potential clinically significant interaction (Amber)”.

**Table 4:** Severity level of DDIs associated with comedications

	Overall		SOF/VEL		GLE/PIB	
Total	7,338		467		6,871	
DDIs: Contraindication (Red)	115	1.6%	0	0.0%	115	1.7%
DDIs: Potential clinically significant interaction (Amber)	2337	31.8%	276	59.1%	2061	30.0%
DDIs: weak interaction (Yellow)	1367	18.6%	1	0.2%	1366	19.9%
No DDIs	3519	48.0%	190	40.7%	3329	48.5%

The denominators for percentage calculation were the total number of cases in each group

Table 5 provides details of comedications classified as “Contraindication (Red)” for GLE/PIB groups, respectively. We identified atorvastatin in 36 patients (0.5%), carbamazepine in 29 patients (0.4%), dabigatran and phenytoin, each in 13 patients (0.2%), and simvastatin in 7 patients (0.1%). Eltrombopag, norethisterone (norethindrone)/ethinyl estradiol, primidone, and desogestrel/ethinyl estradiol were also identified in three or fewer patients.

**Table 5:** Details of comedications classified as “contraindication (Red)” for GEL/PIB group

	GLE/PIB	
Total	6,871	
Atazanavir alone	0	0.0%
Atorvastatin	36	0.5%
Aliskiren	0	0.0%
Amobarbital	1	0.0%
Apalutamide	0	0.0%
Bosentan	2	0.0%
Carbamazepine	29	0.4%
Dabigatran	13	0.2%
Darunavir/cobicistat/FTC/TAF	0	0.0%
Darunavir/cobicistat	0	0.0%
Desogestrel/ethinylestradiol (COC)	0	0.0%
Drospirenone/ethinyl estradiol (COC)	2	0.0%
Efavirenz	0	0.0%
Eltrombopag	3	0.0%
Ethinyl estradiol	0	0.0%
Etravirine	0	0.0%
Fosamprenavir	0	0.0%
Ledipasvir/Sofosbuvir	2	0.0%
Levonorgestrel/ethinyl estradiol (COC)	0	0.0%
Lopinavir	0	0.0%
Nevirapine	0	0.0%
Nirmatrelvir/ritonavir	0	0.0%
Norethisterone (Norethindrone)/ethinyl estradiol (COC)	3	0.0%
Norgestrel/ethinyl estradiol (COC)	0	0.0%
Phenobarbital	10	0.1%
Phenytoin	13	0.2%
Primidone	2	0.0%
Rifabutin	0	0.0%
Rifampicin	0	0.0%
Ritonavir	0	0.0%
Simvastatin	7	0.1%
Sofosbuvir/Velpatasvir	0	0.0%
Vinblastine	0	0.0%
Vincristine	2	0.0%

The denominators for percentage calculation were the total number of cases in GLE/PIB group

Table 6 presents the results of DDI risk for comedications based on common comorbidities and HCV severity. Patients with comorbidities were more likely to be prescribed medications with DDI risk compared to those without comorbidities. For HCV severity, patients with decompensated cirrhosis had a higher proportion of DDI risk compared to those with chronic hepatitis C (65.1% vs. 51.4%). However, among patients in the SOF/VEL group, the difference in the proportion of those with two or more DDIs based on HCV severity was relatively small (13.0% vs. 14.7%).

**Table 6:** DDIs associated with comedications stratified by common comorbidities and HCV severity

			Overall		SOF/VEL		GLE/PIB	
Congestive heart failure	With	Total	1,382		111		1,271	
		DDIs	1,045	75.6%	75	67.6%	970	76.3%
		DDIs > = 2 medications	659	47.7%	30	27.0%	629	49.5%
	Without	Total	5,956		356		5,600	
		DDIs	2,774	46.6%	202	56.7%	2,572	45.9%
		DDIs > = 2 medications	1,055	17.7%	36	10.1%	1,019	18.2%
Cerebrovascular disease	With	Total	1,079		62		1,017	
		DDIs	767	71.1%	40	64.5%	727	71.5%
		DDIs > = 2 medications	435	40.3%	13	21.0%	422	41.5%
	Without	Total	6,259		405		5,854	
		DDIs	3,052	48.8%	237	58.5%	2,815	48.1%
		DDIs > = 2 medications	1,279	20.4%	53	13.1%	1,226	20.9%
Chronic pulmonary disease	With	Total	1,280		94		1,186	
		DDIs	842	65.8%	59	62.8%	783	66.0%
		DDIs > = 2 medications	432	33.8%	15	16.0%	417	35.2%
	Without	Total	6,058		373		5,685	
		DDIs	2,977	49.1%	218	58.4%	2,759	48.5%
		DDIs > = 2 medications	1,282	21.2%	51	13.7%	1,231	21.7%
Peptic ulcer disease	With	Total	1,491		145		1,346	
		DDIs	1,053	70.6%	113	77.9%	940	69.8%
		DDIs > = 2 medications	519	34.8%	22	15.2%	497	36.9%
	Without	Total	5,847		322		5,525	
		DDIs	2,766	47.3%	164	50.9%	2,602	47.1%
		DDIs > = 2 medications	1,195	20.4%	44	13.7%	1,151	20.8%
Any malignancy,	With	Total	1,612		190		1,422	
including lymphoma and leukemia,		DDIs	994	61.7%	119	62.6%	875	61.5%
except malignant neoplasm of skin		DDIs > = 2 medications	444	27.5%	29	15.3%	415	29.2%
	Without	Total	5,726		277		5,449	
		DDIs	2,825	49.3%	158	57.0%	2,667	48.9%
		DDIs > = 2 medications	1,270	22.2%	37	13.4%	1,233	22.6%
Type of HCV	Decompensated cirrhosis	Total	149		129		20	
		DDIs	97	65.1%	84	65.1%	13	65.0%
		DDIs > = 2 medications	27	18.1%	19	14.70%	8	40.0%
	Compensated cirrhosis	Total	234		10		224	
		DDIs	131	56.0%	4	40.0%	127	56.7%
		DDIs > = 2 medications	64	27.4%	0	0.0%	64	28.6%
	Chronic hepatitis C	Total	5,963		247		5,716	
		DDIs	3,067	51.4%	137	55.5%	2,930	51.3%
		DDIs > = 2 medications	1,377	23.1%	32	13.0%	1,345	23.5%
	Others	Total	992		81		911	
		DDIs	524	52.8%	52	64.2%	472	51.8%
		DDIs > = 2 medications	246	24.8%	15	18.5%	231	25.4%

The denominators for percentage calculation were the total number of cases in each subgroup

Table 7 shows the details and proportions of specific comedications. For antipsychotics/neuroleptics, the SOF/VEL group had one drug with a “Weak interaction(Yellow)” DDI risk, involving 4 cases (0.9%) of Risperidone. In contrast, the GLE/PIB group had three drugs classified as “Potential clinically significant interaction (Amber)” with 31 cases (0.5%) of Quetiapine, 20 cases (0.3%) of Aripiprazole, and 10 cases (0.1%) of Paliperidone, respectively. Additionally, two drugs in the GLE/PIB group were categorized as “Weak interaction (Yellow)” involving 57 cases (0.8%) of Risperidone and 1 case (0.0%) of Lurasidone, respectively. For gastrointestinal drugs, the SOF/VEL group had nine drugs with a “Potential clinically significant interaction (Amber)” DDI risk, involving 77 cases (16.5%) of Esomeprazole, 53 cases (11.3%) of Vonoprazan, 46 cases (9.9%) of Lansoprazole, 40 cases (8.6%) of Rabeprazole, 29 cases (6.2%) of Famotidine, 7 cases (1.5%) of Omeprazole, and one case each (0.2%) of Lafutidine, Cimetidine and Ranitidine hydrochloride for three additional drugs. In the GLE/PIB group, three drugs were categorized as “Potential clinically significant interaction (Amber)” involving 88 cases (1.3%) of Domperidone, 37 cases (0.5%) of Sulfasalazine, and 2 cases (0.0%) of Droperidol, respectively. Additionally, twelve drugs in the GLE/PIB group were categorized as “Weak interaction (Yellow)” involving 507 cases (7.4%) of Lansoprazole, 461 cases (6.7%) of Esomeprazole, 359 cases (5.2%) of Vonoprazan, 335 cases (4.9%) of Famotidine, 253 cases (3.7%) of Rabeprazole, 79 cases (1.1%) of Omeprazole, 39 cases (0.6%) of Lafutidine, 37 cases (0.5%) of Loperamide, 23 cases (0.3%) of Cimetidine, 21 cases (0.3%) of Nizatidine, 7 cases (0.1%) of Granisetron, and 5 cases (0.1%) of Roxatidine, respectively. For lipid-lowering agents, three drugs in the SOF/VEL group had a DDI risk classified as “Potential clinically significant interaction (Amber)” involving 5 cases (1.1%) of Atorvastatin, 4 cases (0.9%) of Rosuvastatin, and 1 case (0.2%) of Fluvastatin. In the GLE/PIB group, two drugs were categorized as “Contraindication (Red)” involving 36 cases (0.5%) of Atorvastatin and 7 cases (0.1%) of Simvastatin, while five drugs had a “Potential clinically significant interaction (Amber)” DDI risk, with 163 cases (2.4%) of Rosuvastatin, 67 cases (1.0%) of Pravastatin, 66 cases (1.0%) of Ezetimibe, 66 cases (1.0%) of Pitabastatin, and 5 cases (0.1%) of Fluvastatin, respectively. All comedications with a DDI risk for any DAA drug prescribed during the treatment period are detailed in Supplementary Table 1. Comedications classified as contraindicated in Liverpool HEP interaction checker or Japanese package inserts are detailed in Supplementary Table 2 (SOV/VEL in Table S2a; GLE/PIB in Table S2b).

**Table 7:**
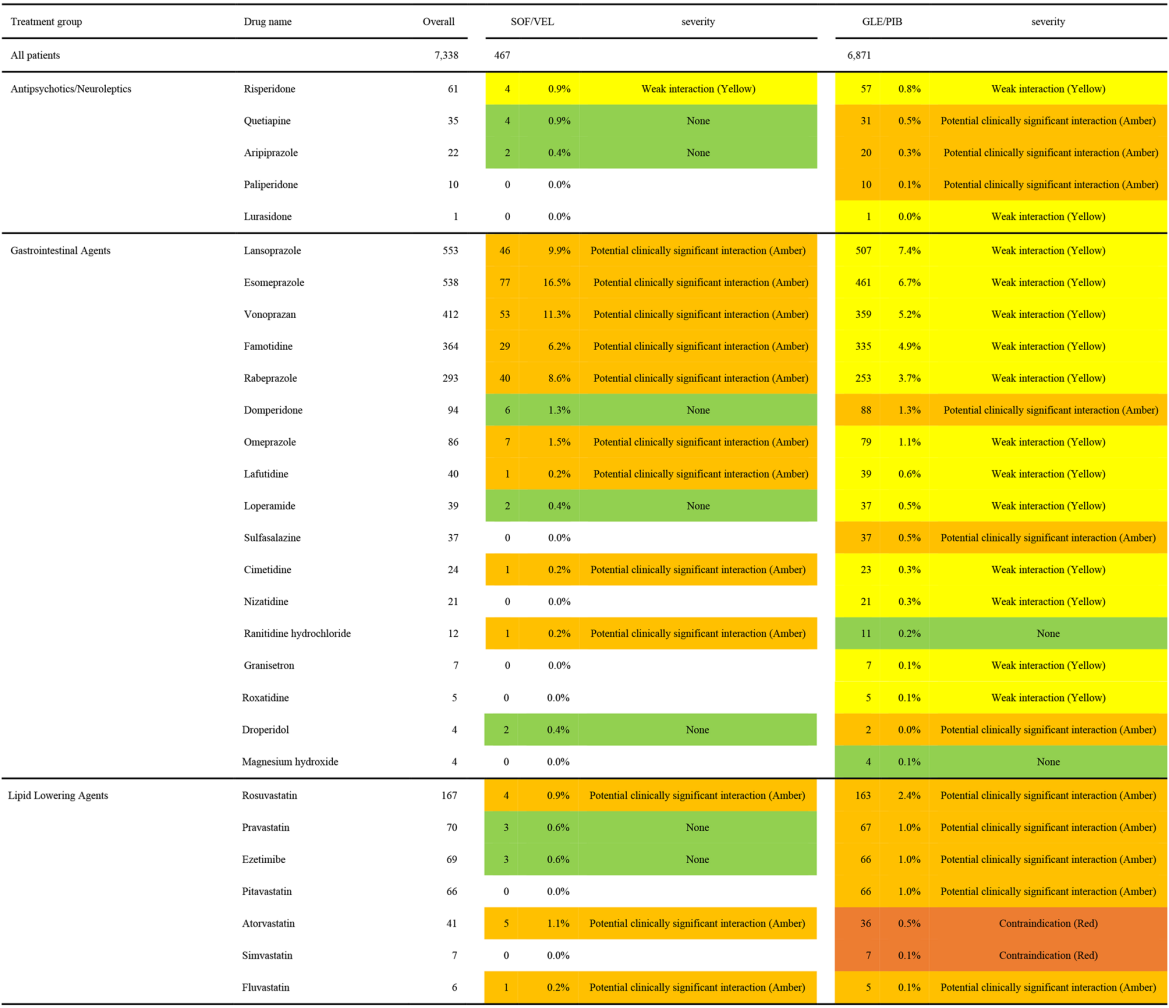
Details of specific comedications and their proportions

## Discussion

This study showed that over half of the HCV patients who received DAA therapy were prescribed comedications with DDI risk during the treatment. Most patients who were prescribed comedications with DDI risk fell into the categories of “Potential clinically significant interaction (Amber)” or “Weak interaction (Yellow)”, however, a few patients in the GLE/PIB group were classified as “Contraindication (Red)”. Among the comedications classified as “Contraindication (Red)” for DDI risk, medications such as simvastatin, dabigatran and eltrombopag were included. While these medications are not listed in the package inserts, they were flagged by the Liverpool HEP checker. When evaluating DDIs, it is important to understand the metabolic pathways of the target drug. However, Japanese package inserts sometimes lack sufficient information about the specific enzyme species involved in metabolism. Naturally, details regarding the metabolic contribution rates of particular enzyme species are rarely provided. Furthermore, updates to DDI information in product package inserts are infrequent after a drug launch, which may cause such discrepancies. On the other hand, in Western countries, guidelines from EASL and AASLD recommend the use of external platforms, such as the Liverpool HEP Checker, to bridge this gap and comprehensively evaluate DDIs [14, 15].

In this study, the proportion of patients who were prescribed comedications with DDI risk was 52% (59.3% in SOF/VEL, 51.5% in GLE/PIB), with 23% (14.1% in SOF/VEL, 24.0% in GLE/PIB) receiving two or more DDI risk medications. These proportions were relatively higher compared to those previously reported. An Italian observational study evaluating DDI risk during DAA therapy in HCV patients, similar to current study, reported a DDI risk proportion of 28.7% in the SOF/VEL group and 20.9% in the GLE/PIB group [16]. Another Italian study showed the proportion of patients at risk for two or more DDIs was 11.6% in the SOF/VEL group and 19.6% in the GLE/PIB group [14]. The relatively higher proportion of patients with DDI risk in our study is likely due to the older age and higher comorbidity burden in the Japanese HCV population. Indeed, in the previous studies, the mean patient age was in the 50s with a mean CCI score of less than 1, whereas in our study, the mean age was 69.9 years, and the median CCI score was 3.0.

A relatively higher proportion of patients with DDI risk was observed in the SOF/VEL group (59.3%) compared to the GLE/PIB group (51.5%) in this study, which is likely due to differences in patient characteristics between the groups including age, comorbidities, and disease severity. The SOF/VEL group included approximately 30% of patients with decompensated cirrhosis, as SOF/VEL is indicated for this condition.

According to clinical guidelines, NS3/4A protease inhibitors, including paritaprevir, grazoprevir, glecaprevir, and voxilaprevir are contraindicated in patients with Child–Pugh class B or C decompensated cirrhosis due to significant elevation in protease inhibitor concentrations and the associated risk of toxicity [12, 22]. In Japan, SOF/VEL is the only approved treatment for patients with decompensated cirrhosis. Additionally, patients in the SOF/VEL group were older, had more comorbidities, and were on more baseline medications than those in the GLE/PIB group. While current study and previous research [16] have consistently shown a higher proportion of patients with DDI risk in the SOF/VEL group compared to the GLE/PIB group, other studies have reported the opposite, with a higher DDI risk in the GLE/PIB group [15, 21]. This inconsistency may be due to regional differences in drug usage, patient populations, or variations in the methodologies used to assess DDI risks. Previous studies examining potential DDIs in elderly and younger patients have shown that the frequency of DDIs is higher in elderly patients [25]. In this study, the fact that SOF/VEL was more frequently prescribed to elderly patients at baseline may have influenced the results.

In the GLE/PIB group, 24% of patients had two or more DDIs, which was higher than the 14% observed in the SOF/VEL group. Recent Spanish and Italian studies using electronic health record (EMR) data and administrative databases reported fewer multi-DDIs in the SOF/VEL group, consistent with our findings [14, 26]. The drugs contributing to DDI risk in the SOF/VEL group were primarily antacids, H2 blockers, and proton pump inhibitors (supplement Table 1), which have a potential for interaction but pose no significant risk when used at standard doses [27]. It was considered that many of the patients in the SOF/VEL group with DDI risk were exposed to these drugs as single agents. Even among patients with decompensated cirrhosis in the SOF/VEL group, the proportion of those with two or more DDIs was 14.7%, similar to the overall results. Since the launch of ledipasvir/sofosbuvir in Japan in 2015, education on the appropriate use of sofosbuvir-containing regimens with proton pump inhibitors (PPIs) or H2 blockers. In contrast, 1.7% of patients in the GLE/PIB group were found to have medications classified as “Contraindication (Red),” highlighting the presence of high DDI risk. In a previous Italian study, 0.4% of patients in the SOF/VEL group and 3.2% in the GLE/PIB group were identified as having “Contraindication (Red)” interactions [16]. Similarly, a Spanish study reported 1.7% for SOF/VEL and 8.3% for GLE/PIB in this category [15]. The Liverpool HEP checker, used in this and previous studies, includes not only drugs listed as contraindicated in Japanese package inserts but also similar drugs with the same mechanism of action. As a result, this study also found a higher proportion of potential DDIs in the GLE/PIB group (28.5%) compared to the SOF/VEL group (14.8%). Medications such as carbamazepine, dabigatran, and phenytoin, which are not explicitly listed in the package insert but flagged by the HEP checker, were identified as “Contraindication.” This highlighted the need to consider potential DDI risks, including those flagged by the Liverpool HEP checker.

This study builds on previous findings regarding DDI risks associated with DAA therapy by highlighting the real-world settings in an aging Japanese population, where polypharmacy is a significant concern. A previous study using Japan’s Medical Data Vision (MDV) database, which includes inpatient, outpatient, and pharmacy claims as well as diagnosis-procedure combination data, evaluated DDI risks prior to DAA therapy for chronic hepatitis C patients based on data up to 2016. The MDV database, being hospital-based, captures data only from institutions where patients receive DAA treatment [4]. In contrast, this study used the insurance-based DeSC database, which includes medical records from clinics and other facilities outside the DAA treatment centers, allowing for a more comprehensive evaluation of DDI risk. The results suggest that older patients with multiple comorbidities are at higher risk of being prescribed comedications with potential DDI risk during DAA therapy. This highlights the need to monitor not only contraindicated drugs but also potential DDI risks beyond those listed in package inserts. As the aging HCV population is expected to grow globally, this study offers valuable insights into managing DDI risk in older HCV patient populations. However, this study did not evaluate clinical outcomes, such as adverse events related to DDIs. Future research is needed to fully understand the real-world implications of DDI risks.

There are several limitations in this study. First, since the database used in this research is derived from claims records, it does not include the values of clinical laboratory results or disease severities, thereby precluding the collection of detailed clinical information and pharmacokinetic parameters such as changes in the area under the curve. As a result, the impact of liver function on DDI risk could not be assessed. Another limitation is that the DeSC database is insurance-based, which means that patients are tracked only during their enrollment period. Additionally, if patients switch insurance, they may be counted as separate individuals. Despite these challenges, the curative nature of DAA therapy, with rare cases of retreatment, reduces the risk of double-counting within the study cohort. Moreover, setting a 90-day look-back period improves accuracy in identifying new DAA users, further minimizing the chance of duplicate patient inclusion. Third, the database does not collect information on the DAA treatment duration predetermined by the physician as part of the therapeutic strategy. Therefore, it is impossible to determine whether the treatment end date in the study reflects actual completion or discontinuation. Fourth, this study assessed DDI risk based on comedications prescribed during the DAA treatment period rather than those prescribed before DAA initiation. This approach aligned with the study’s focus but may have underestimated actual use because comedications prescribed before DAA initiation and continued afterward were not included in the calculation.

## Conclusion

This study investigated the real-world prescription patterns of comedications associated with DDI risk of comedications in HCV patients undergoing DAA therapy using a large Japanese database that included older adults. A considerable proportion of patients were prescribed medications with DDI risk during DAA treatment. A small but notable proportion of patients were on “Contraindication (Red)” medications. Consideration of the potential DDI risks associated with comedications by healthcare professionals is advised, referring not only to package inserts but also tools such as the Liverpool HEP checker to guide safe prescribing when initiating DAA therapy for HCV patients.

## Electronic supplementary material

Below is the link to the electronic supplementary material.

Supplementary Material 1

## Data Availability

The datasets generated during and/or analyzed during the current study are not publicly available due to the research contracts with the data suppliers.

## Abbreviations

DAA: Direct Acting Antivirals
SVR: Sustained virologic response
CHC: Chronic hepatitis C virus
SOF/VEL: Sofosbuvir /Velpatasvir
GLE/PIB: Glecaprevir/Pibrentasvir
DDIs: Drug-drug interaction
HCPs: Healthcare providers
AASLD: The American Association for the Study of Liver Diseases
Liverpool HEP Checker: The European Association for the Study of the Liver: EASLLiverpool University HEP Drug Interaction Checker tool
JSH: The Japan Society of Hepatology
WHO: World Health Organization
SHI = Kenpo: Society-managed, employment-based health insurance association
NHI = Kokuho: National Health Insurance
LSEHS = Koki Koreisha Iryo Seido: Latter-Stage Elderly Healthcare System
ATC: Anatomical Therapeutic Chemical
CCI: Charlson Comorbidity Index
MDV: Medical Data Vision
EMR: Electronic health record
PPIs: Proton pump inhibitors
