# Supplementary material for: Prescription patterns of comedications associated with drug-drug interactions risk in HCV-infected patients undergoing direct-acting antiviral treatment: an analysis of an administrative claims database in Japan
Source: J Pharm Health Care Sci. 2025 Apr 18;11:33. doi: 10.1186/s40780-025-00442-5 (PMC12008892; doi:10.1186/s40780-025-00442-5)
Supplement: Supplementary file 1 — Supplementary Material 1 [file 40780_2025_442_MOESM1_ESM.docx]

**Supplementary Information**

**Article title**

Prescription patterns of comedications associated with drug-drug interactions risk in HCV-infected patients undergoing direct-acting antiviral treatment: An analysis of an administrative claims database in Japan

**Author & Affiliations**

Daisuke Nakamoto^1^, Yi Piao^1^, Hajime Mizutani^1^, Ryozo Wakabayashi^2^, Satoshi Otokita^2^, Alice Stead^3^, Candido Hernandez^3^, Masahisa Jinushi^1^

1 Gilead Sciences K.K., 1-9-2 Marunochi Gran Tokyo South Tower 16F, Chiyoda-ku, Tokyo 100-6616, Japan

2 Datack, Inc., 707, 1-8-9, Iidabashi Chiyoda-ku, Tokyo 102-0072, Japan

3 Gilead Sciences, Inc., 333 Lakeside Drive Foster City, CA 94404, USA

**Supplementary tables**

Table S1. Details of comedications and their proportions (Contraindication, Weak interaction, Potential clinically significant interaction)

| Treatment group | Drug name | Overall |  | SOF/VEL | | severity |  | GLE/PIB | | severity |
| --- | --- | --- | --- | --- | --- | --- | --- | --- | --- | --- |
| All patients |  | 7,338 |  | 467 |  |  |  | 6,871 |  |  |
| Analgesics | Fentanyl (Prescribed) | 24 |  | 4 | 0.9% | None |  | 20 | 0.3% | Potential clinically significant interaction |
|  | Oxycodone | 6 |  | 1 | 0.2% | None |  | 5 | 0.1% | Potential clinically significant interaction |
| Antiarrhythmics | Amiodarone | 15 |  | 0 | 0.0% |  |  | 15 | 0.2% | Potential clinically significant interaction |
|  | Digoxin | 27 |  | 2 | 0.4% | Potential clinically significant interaction |  | 25 | 0.4% | Potential clinically significant interaction |
| Antibacterials | Clarithromycin | 119 |  | 2 | 0.4% | None |  | 117 | 1.7% | Weak interaction |
|  | Erythromycin | 9 |  | 1 | 0.2% | None |  | 8 | 0.1% | Potential clinically significant interaction |
|  | Isoniazid | 5 |  | 0 | 0.0% |  |  | 5 | 0.1% | Weak interaction |
| Antibacterials | Rifaximin | 52 |  | 46 | 9.9% | None |  | 6 | 0.1% | Potential clinically significant interaction |
| Anticoagulant, Anti-platelet and Fibrinolytic | Apixaban | 87 |  | 7 | 1.5% | Potential clinically significant interaction |  | 80 | 1.2% | Potential clinically significant interaction |
|  | Dabigatran | 14 |  | 1 | 0.2% | Potential clinically significant interaction |  | 13 | 0.2% | Contraindication |
|  | Edoxaban | 131 |  | 15 | 3.2% | Potential clinically significant interaction |  | 116 | 1.7% | Potential clinically significant interaction |
|  | Eltrombopag | 4 |  | 1 | 0.2% | Weak interaction |  | 3 | 0.0% | Contraindication |
|  | Rivaroxaban | 54 |  | 3 | 0.6% | Potential clinically significant interaction |  | 51 | 0.7% | Potential clinically significant interaction |
|  | Warfarin | 109 |  | 8 | 1.7% | Potential clinically significant interaction |  | 101 | 1.5% | Potential clinically significant interaction |
| Anticonvulsants | Carbamazepine | 29 |  | 0 | 0.0% |  |  | 29 | 0.4% | Contraindication |
|  | Phenobarbital | 10 |  | 0 | 0.0% |  |  | 10 | 0.1% | Contraindication |
|  | Phenytoin | 13 |  | 0 | 0.0% |  |  | 13 | 0.2% | Contraindication |
|  | Primidone | 2 |  | 0 | 0.0% |  |  | 2 | 0.0% | Contraindication |
|  | Mianserin | 11 |  | 0 | 0.0% |  |  | 11 | 0.2% | Weak interaction |
| Antidiabetics | Dulaglutide | 42 |  | 5 | 1.1% | Weak interaction |  | 37 | 0.5% | None |
|  | Empagliflozin | 53 |  | 2 | 0.4% | Potential clinically significant interaction |  | 51 | 0.7% | None |
|  | Glibenclamide (Glyburide) | 10 |  | 1 | 0.2% | None |  | 9 | 0.1% | Potential clinically significant interaction |
|  | Liraglutide | 8 |  | 0 | 0.0% |  |  | 8 | 0.1% | None |
|  | Lixisenatide | 1 |  | 0 | 0.0% |  |  | 1 | 0.0% | None |
|  | Repaglinide | 44 |  | 0 | 0.0% |  |  | 44 | 0.6% | Potential clinically significant interaction |
|  | Semaglutide | 8 |  | 0 | 0.0% |  |  | 8 | 0.1% | None |
|  | Vildagliptin | 79 |  | 6 | 1.3% | None |  | 73 | 1.1% | Weak interaction |
| Antifungals | Ketoconazole | 46 |  | 7 | 1.5% | None |  | 39 | 0.6% | Potential clinically significant interaction |
| Antihistamines | Bilastine | 120 |  | 10 | 2.1% | Potential clinically significant interaction |  | 110 | 1.6% | Weak interaction |
|  | Ebastine | 24 |  | 0 | 0.0% |  |  | 24 | 0.3% | Potential clinically significant interaction |
|  | Fexofenadine | 330 |  | 12 | 2.6% | None |  | 318 | 4.6% | Weak interaction |
| Antipsychotics/Neuroleptics | Aripiprazole | 22 |  | 2 | 0.4% | None |  | 20 | 0.3% | Potential clinically significant interaction |
|  | Lurasidone | 1 |  | 0 | 0.0% |  |  | 1 | 0.0% | Weak interaction |
|  | Paliperidone | 10 |  | 0 | 0.0% |  |  | 10 | 0.1% | Potential clinically significant interaction |
|  | Quetiapine | 35 |  | 4 | 0.9% | None |  | 31 | 0.5% | Potential clinically significant interaction |
|  | Risperidone | 61 |  | 4 | 0.9% | Weak interaction |  | 57 | 0.8% | Weak interaction |
| Anxiolytics/Hypnotics/Sedatives | Amobarbital | 1 |  | 0 | 0.0% |  |  | 1 | 0.0% | Contraindication |
| Beta Blockers | Carvedilol | 265 |  | 6 | 1.3% | Potential clinically significant interaction |  | 259 | 3.8% | Potential clinically significant interaction |
| Bronchodilators | Theophylline | 39 |  | 2 | 0.4% | None |  | 37 | 0.5% | Potential clinically significant interaction |
| Calcium Channel Blockers | Diltiazem | 63 |  | 0 | 0.0% |  |  | 63 | 0.9% | Potential clinically significant interaction |
|  | Verapamil | 56 |  | 3 | 0.6% | None |  | 53 | 0.8% | Potential clinically significant interaction |
| Cancer Therapies | Doxorubicin | 1 |  | 0 | 0.0% |  |  | 1 | 0.0% | Potential clinically significant interaction |
|  | Enzalutamide | 8 |  | 0 | 0.0% |  |  | 8 | 0.1% | Potential clinically significant interaction |
|  | Everolimus | 1 |  | 0 | 0.0% |  |  | 1 | 0.0% | Potential clinically significant interaction |
|  | Methotrexate | 35 |  | 1 | 0.2% | Potential clinically significant interaction |  | 34 | 0.5% | Potential clinically significant interaction |
|  | Nilotinib | 1 |  | 0 | 0.0% |  |  | 1 | 0.0% | Potential clinically significant interaction |
|  | Tamoxifen | 9 |  | 1 | 0.2% | Weak interaction |  | 8 | 0.1% | None |
|  | Vincristine | 2 |  | 0 | 0.0% |  |  | 2 | 0.0% | Contraindication |
| Contraceptives & HRT | Drospirenone/ethinylestradiol (COC) | 2 |  | 0 | 0.0% |  |  | 2 | 0.0% | Contraindication |
|  | Norethisterone (Norethindrone)/ethinylestradiol (COC) | 3 |  | 0 | 0.0% |  |  | 3 | 0.0% | Contraindication |
| Gastrointestinal Agents | Cimetidine | 24 |  | 1 | 0.2% | Potential clinically significant interaction |  | 23 | 0.3% | Weak interaction |
|  | Domperidone | 94 |  | 6 | 1.3% | None |  | 88 | 1.3% | Potential clinically significant interaction |
|  | Droperidol | 4 |  | 2 | 0.4% | None |  | 2 | 0.0% | Potential clinically significant interaction |
|  | Esomeprazole | 538 |  | 77 | 16.5% | Potential clinically significant interaction |  | 461 | 6.7% | Weak interaction |
|  | Famotidine | 364 |  | 29 | 6.2% | Potential clinically significant interaction |  | 335 | 4.9% | Weak interaction |
|  | Granisetron | 7 |  | 0 | 0.0% |  |  | 7 | 0.1% | Weak interaction |
|  | Lafutidine | 40 |  | 1 | 0.2% | Potential clinically significant interaction |  | 39 | 0.6% | Weak interaction |
|  | Lansoprazole | 553 |  | 46 | 9.9% | Potential clinically significant interaction |  | 507 | 7.4% | Weak interaction |
|  | Loperamide | 39 |  | 2 | 0.4% | None |  | 37 | 0.5% | Weak interaction |
|  | Magnesium hydroxide | 4 |  | 0 | 0.0% |  |  | 4 | 0.1% | None |
|  | Nizatidine | 21 |  | 0 | 0.0% |  |  | 21 | 0.3% | Weak interaction |
|  | Omeprazole | 86 |  | 7 | 1.5% | Potential clinically significant interaction |  | 79 | 1.1% | Weak interaction |
|  | Rabeprazole | 293 |  | 40 | 8.6% | Potential clinically significant interaction |  | 253 | 3.7% | Weak interaction |
|  | Ranitidine hydrochloride | 12 |  | 1 | 0.2% | Potential clinically significant interaction |  | 11 | 0.2% | None |
|  | Roxatidine | 5 |  | 0 | 0.0% |  |  | 5 | 0.1% | Weak interaction |
|  | Sulfasalazine | 37 |  | 0 | 0.0% |  |  | 37 | 0.5% | Potential clinically significant interaction |
|  | Vonoprazan | 412 |  | 53 | 11.3% | Potential clinically significant interaction |  | 359 | 5.2% | Weak interaction |
| Hepatitis C Drugs | Ledipasvir/Sofosbuvir | 2 |  | 0 | 0.0% |  |  | 2 | 0.0% | Contraindication |
| HIV Entry/Integrase Inhibitors | Bictegravir/FTC/TAF | 2 |  | 0 | 0.0% |  |  | 2 | 0.0% | Weak interaction |
| Hypertension/Heart Failure Agents | Bosentan | 2 |  | 0 | 0.0% |  |  | 2 | 0.0% | Contraindication |
|  | Candesartan | 274 |  | 12 | 2.6% | None |  | 262 | 3.8% | Weak interaction |
|  | Enalapril | 85 |  | 2 | 0.4% | None |  | 83 | 1.2% | Potential clinically significant interaction |
|  | Eplerenone | 32 |  | 1 | 0.2% | None |  | 31 | 0.5% | Potential clinically significant interaction |
|  | Irbesartan | 74 |  | 4 | 0.9% | None |  | 70 | 1.0% | Potential clinically significant interaction |
|  | Olmesartan | 324 |  | 21 | 4.5% | None |  | 303 | 4.4% | Potential clinically significant interaction |
|  | Prazosin | 2 |  | 0 | 0.0% |  |  | 2 | 0.0% | Potential clinically significant interaction |
|  | Sacubitril/valsartan | 21 |  | 3 | 0.6% | None |  | 18 | 0.3% | Potential clinically significant interaction |
|  | Telmisartan | 231 |  | 8 | 1.7% | None |  | 223 | 3.2% | Potential clinically significant interaction |
| Illicit/Recreational | Etizolam | 314 |  | 10 | 2.1% | None |  | 304 | 4.4% | Potential clinically significant interaction |
| Immunosuppressants | Ciclosporin | 8 |  | 1 | 0.2% | None |  | 7 | 0.1% | Potential clinically significant interaction |
|  | Tacrolimus | 43 |  | 3 | 0.6% | Potential clinically significant interaction |  | 40 | 0.6% | Potential clinically significant interaction |
| Lipid Lowering Agents | Atorvastatin | 41 |  | 5 | 1.1% | Potential clinically significant interaction |  | 36 | 0.5% | Contraindication |
|  | Ezetimibe | 69 |  | 3 | 0.6% | None |  | 66 | 1.0% | Potential clinically significant interaction |
|  | Fluvastatin | 6 |  | 1 | 0.2% | Potential clinically significant interaction |  | 5 | 0.1% | Potential clinically significant interaction |
|  | Pitavastatin | 66 |  | 0 | 0.0% |  |  | 66 | 1.0% | Potential clinically significant interaction |
|  | Pravastatin | 70 |  | 3 | 0.6% | None |  | 67 | 1.0% | Potential clinically significant interaction |
|  | Rosuvastatin | 167 |  | 4 | 0.9% | Potential clinically significant interaction |  | 163 | 2.4% | Potential clinically significant interaction |
|  | Simvastatin | 7 |  | 0 | 0.0% |  |  | 7 | 0.1% | Contraindication |
| Steroids | Dexamethasone >16 mg | 112 |  | 10 | 2.1% | Potential clinically significant interaction |  | 102 | 1.5% | None |
| Urological Agents | Mirabegron | 95 |  | 5 | 1.1% | None |  | 90 | 1.3% | Potential clinically significant interaction |
| Urological Agents | Silodosin | 113 |  | 9 | 1.9% | Potential clinically significant interaction |  | 104 | 1.5% | Potential clinically significant interaction |
| Urological Agents | Solifenacin | 34 |  | 2 | 0.4% | None |  | 32 | 0.5% | Weak interaction |
| Other | Activated charcoal | 19 |  | 1 | 0.2% | Weak interaction |  | 18 | 0.3% | Weak interaction |
|  | Colchicine | 5 |  | 0 | 0.0% |  |  | 5 | 0.1% | Potential clinically significant interaction |
|  | Guanfacine | 1 |  | 0 | 0.0% |  |  | 1 | 0.0% | Weak interaction |
|  | Sevelamer | 27 |  | 0 | 0.0% |  |  | 27 | 0.4% | Weak interaction |
| The denominators for percentage calculation were the total number of cases in each group | |  |  |  |  |  |  |  |  |  |

Table S2a. Comedications classified as contraindicated for SOF/VEL in Liverpool HEP interaction checker or Japanese package inserts

|  | Mechanisms of interaction | listed in the JPI |
| --- | --- | --- |
| Carbamazepine | Coadministration has not been formally studied and is not recommended. Concentrations of sofosbuvir/velpatasvir may decrease due to induction of P-gp and/or CYPs 2B6, 2C8, and 3A4 by carbamazepine; this may lead to reduced therapeutic effect and potential virological failure. Note - there are a small number of cases reported of patients who have remained on an inducing antiepileptic during HCV DAA therapy (including with sofosbuvir/velpatasvir) and achieved a sustained virologic response. Although every effort to prevent concomitant use of sofosbuvir/velpatasvir and potent inducers should be made, these cases have demonstrated that clinical cure may still be achieved in patients where coadministration cannot be avoided. Therapeutic drug level monitoring could be considered, where available. | ✓ |
| Phenobarbital | Coadministration has not been formally studied and is not currently recommended. Concentrations of sofosbuvir and velpatasvir may decrease due to induction of P-gp and/or CYPs 2B6, 2C8 and 3A4 by phenobarbital; this may lead to reduced therapeutic effect and potential virological failure. Note - there are a small number of cases reported of patients who have remained on an inducing antiepileptic during HCV DAA therapy (including with sofosbuvir/velpatasvir) and achieved a sustained virologic response. Although every effort to prevent concomitant use of sofosbuvir/velpatasvir and potent inducers should be made, these cases have demonstrated that clinical cure may still be achieved in patients where coadministration cannot be avoided. Therapeutic drug level monitoring could be considered, where available. | ✓ |
| Phenytoin | Coadministration has not been formally studied and is not currently recommended. Concentrations of velpatasvir and sofosbuvir may decrease due to induction of P-gp and/or CYPs 2B6, 2C8 and 3A4 by phenytoin; this may lead to reduced therapeutic effect and potential virological failure. Note - there are a small number of cases reported of patients who have remained on an inducing antiepileptic during HCV DAA therapy (including with sofosbuvir/velpatasvir) and achieved a sustained virologic response. Although every effort to prevent concomitant use of sofosbuvir/velpatasvir and potent inducers should be made, these cases have demonstrated that clinical cure may still be achieved in patients where coadministration cannot be avoided. Therapeutic drug level monitoring could be considered, where available | ✓ |
| Rifampicin | Coadministration is contraindicated as concentrations of velpatasvir and sofosbuvir are significantly decreased. This may result in loss of efficacy and potential virological failure. Coadministration with rifampicin (600 mg once daily) decreased Cmax and AUC of sofosbuvir by 77% and 72%, and Cmax and AUC of velpatasvir by 71% and 82%. The effect on rifampicin exposure was not studied but no change is expected. | ✓ |
| Amiodarone | Coadministration has not been studied and is not recommended. Coadministration of amiodarone and sofosbuvir combined with another direct acting antiviral such as velpatasvir may result in serious symptomatic bradycardia. The mechanism of this effect is unknown. The effect on amiodarone, sofosbuvir, and velpatasvir concentrations unknown. If coadministration is required as no other alternative is available, cardiac monitoring is recommended. |  |
| Amobarbital | Coadministration has not been studied and is not recommended. Amobarbital induces CYP3A4 and P-gp and could decrease concentrations of sofosbuvir/velpatasvir. This may result in loss of efficacy and potential virological failure. |  |
| Apalutamide | Coadministration has not been studied and is contraindicated. Sofosbuvir and velpatasvir are substrates of P-gp and BCRP. Velpatasvir is also a substrate of OATP1B and CYP2B6, 2C8 and 3A4. Coadministration may decrease sofosbuvir/velpatasvir concentrations due to induction of CYP3A4 (strong) and P-gp, BCRP and OATP1B1 (weak) by apalutamide; this may lead to reduced therapeutic effect and potential virological failure. [Note, use of apalutamide in severe hepatic impairment is not recommended in the European SmPC.] |  |
| Bosentan | Coadministration has not been studied. Bosentan, a moderate CYP3A inducer, may decrease sofosbuvir/velpatasvir concentrations, leading to reduced therapeutic effect of sofosbuvir/velpatasvir. Coadministration is not recommended. |  |
| Efavirenz | Coadministration of sofosbuvir/velpatasvir with efavirenz-containing regimens is not recommended due to decreased concentrations of velpatasvir. Coadministration of efavirenz (600 mg once daily with emtricitabine and tenofovir-DF) and sofosbuvir/velpatasvir (400/100 mg once daily) increased sofosbuvir Cmax by 38% but decreased AUC by 3%; velpatasvir Cmax, AUC and Cmin decreased by 47%, 53% and 57%, respectively (n=14). Efavirenz Cmax, AUC and Cmin decreased by 19%, 15% and 10%, respectively (n=15). |  |
| Enzalutamide | Coadministration has not been studied and is contraindicated. Sofosbuvir and velpatasvir are substrates of P-gp and BCRP. Velpatasvir is also a substrate of OATP1B and CYP2B6, 2C8 and 3A4. Coadministration may decrease sofosbuvir/velpatasvir concentrations due to induction of CYP3A4, and possibly OATP1B1 by enzalutamide; this may lead to reduced therapeutic effect and potential virological failure. |  |
| Etravirine | Coadministration has not been studied and is not recommended. Concentrations of velpatasvir and sofosbuvir may decrease due to induction of CYP3A4 by etravirine, resulting in loss of efficacy and potential virological failure. |  |
| Glecaprevir/Pibrentasvir | Coadministration of glecaprevir/pibrentasvir with other directly acting antivirals has not been studied and therefore cannot be recommended. |  |
| Ledipasvir/Sofosbuvir | Sofosbuvir/velpatasvir is a complete regimen containing an NS5A. There are no data to support coadministration with other NS5A containing DAAs. |  |
| Modafinil | Coadministration has not been studied and is not recommended. Modafinil, a moderate CYP3A inducer, may decrease sofosbuvir/velpatasvir concentrations, leading to reduced therapeutic effect. |  |
| Nevirapine | Coadministration has not been studied and is not recommended. Nevirapine is an inducer of CYP3A and potentially CYP2B6, with maximal induction occurring within 2-4 weeks of initiating multiple-dose therapy. Coadministration may decrease concentrations of velpatasvir and sofosbuvir due to induction of CYP3A4 and CYP2B6 by nevirapine, resulting in loss of efficacy and potential virological failure. |  |
| Primidone | Coadministration has not been formally studied and is not currently recommended. Both primidone and its major metabolite phenobarbitone are metabolized by, and also induce, liver enzyme activity, principally CYP3A4. Induction of P-gp and CYP3A4 by phenobarbital (a major metabolite of primidone) may significantly decrease plasma concentrations of velpatasvir and sofosbuvir; this may lead to reduced therapeutic effect and potential virological failure. Note - there are a small number of cases reported of patients who have remained on an inducing antiepileptic during HCV DAA therapy (including with sofosbuvir/velpatasvir) and achieved a sustained virologic response. Although every effort to prevent concomitant use of sofosbuvir/velpatasvir and potent inducers should be made, these cases have demonstrated that clinical cure may still be achieved in patients where coadministration cannot be avoided. Therapeutic drug level monitoring could be considered, where available. |  |
| Rifabutin | Coadministration has not been studied and is contraindicated. Concentrations of velpatasvir and sofosbuvir may decrease due to induction of P-gp and/or CYPs 2B6, 2C8 and 3A4 by rifabutin, resulting in loss of efficacy and potential virological failure. | ✓ |
| Sofosbuvir | Sofosbuvir/velpatasvir is a fixed dose combination and should not be administered with other products containing sofosbuvir. |  |

JPI: Japanese package inserts

Table S2b. Comedications classified as contraindicated for GEL/PIB in Liverpool HEP interaction checker or Japanese package inserts

|  | Mechanisms of interaction | listed in the JPI |
| --- | --- | --- |
| Atazanavir alone | Coadministration has not been studied and is contraindicated. Coadministration of glecaprevir/pibrentasvir with OATP1B inhibitors, such as atazanavir, is contraindicated due to increased risk of ALT elevations. Coadministration of atazanavir/ritonavir (300/100 mg once daily) and glecaprevir/pibrentasvir increased glecaprevir Cmax, AUC and Cmin by at least 4.06-fold, 6.53-fold and 14.3-fold, respectively. Pibrentasvir Cmax, AUC and Cmin increased by at least 29%, 64% and 129%, respectively (n=12). | ✓ |
| Atorvastatin | Coadministration of glecaprevir/pibrentasvir and atorvastatin (10 mg single dose) increased atorvastatin AUC by 8.28-fold and increased Cmax by 22-fold. Coadministration is not recommended due to increased levels of atorvastatin, caused by inhibition of OATP1B1, P-gp and BCRP by glecaprevir/pibrentasvir. | ✓ |
| Aliskiren | Coadministration has not been studied. Aliskiren is a substrate of P-gp and concentrations may increase due to inhibition of P-gp by glecaprevir/pibrentasvir. Concomitant use with aliskiren is not recommended. |  |
| Amobarbital | Coadministration has not been studied and is not recommended. Amobarbital induces CYP3A4 and P-gp and could decrease concentrations of glecaprevir/pibrentasvir. This may result in loss of efficacy and potential virological failure. |  |
| Apalutamide | Coadministration has not been studied and is not recommended. Glecaprevir and pibrentasvir are substrates of P-gp and/or BCRP. Glecaprevir is also a substrate of OATP1B1/3. Coadministration may decrease glecaprevir/pibrentasvir concentrations due to induction of P-gp, BCRP, and OATP1B1 (weak) by apalutamide; this may lead to reduced therapeutic effect and potential virological failure. [Note, use of apalutamide in severe hepatic impairment is not recommended in the European SmPC.] |  |
| Bosentan | Coadministration is not recommended. Coadministration of glecaprevir/pibrentasvir with bosentan, a moderate CYP3A inducer, may decrease concentrations of glecaprevir/pibrentasvir, leading to reduced therapeutic effect. |  |
| Carbamazepine | Coadministration is not recommended. Induction of CYP3A4 and/or P-gp by carbamazepine is expected to significantly decrease plasma concentrations of glecaprevir/pibrentasvir; this may lead to reduced therapeutic effect and potential virological failure. Coadministration of glecaprevir/pibrentasvir (single dose) and carbamazepine (200 mg twice daily) decreased glecaprevir Cmax and AUC by 67% and 66% and decreased pibrentasvir Cmax and AUC by 50% and 51%. Note - there are a small number of cases reported of patients who have remained on an inducing antiepileptic during HCV DAA therapy (including with glecaprevir/pibrentasvir) and achieved a sustained virologic response. Although every effort to prevent concomitant use of glecaprevir/pibrentasvir and potent inducers should be made, these cases have demonstrated that clinical cure may still be achieved in patients where coadministration cannot be avoided. Therapeutic drug level monitoring could be considered, where available. |  |
| Dabigatran | Coadministration increased dabigatran exposure due to P-gp inhibition by glecaprevir/pibrentasvir. Coadministration of dabigatran (150 mg single dose) and glecaprevir/pibrentasvir increased dabigatran Cmax and AUC by 2.05-fold and 2.38-fold. Coadministration is contraindicated in the European SPC for glecaprevir/pibrentasvir. However, the US Prescribing Information for glecaprevir/pibrentasvir refers to the dabigatran Prescribing Information which suggests no dose adjustment is needed in subjects with normal renal function, but to reduce dabigatran to 75 mg twice daily in subjects with creatinine clearance 30-50 mL/min (or avoid use) and does not recommend coadministration in subjects with creatinine clearance <30 mL/min. |  |
| Darunavir/cobicistat/FTC/TAF | Coadministration with Symtuza has not been studied and is not recommended as it may substantially increase glecaprevir exposure. Medicinal products that inhibit OATP1B1/3 (e.g. darunavir/cobicistat increase systemic concentrations of glecaprevir. Coadministration of darunavir/ritonavir (800/100 mg) increased glecaprevir AUC, Cmax and Cmin by 4.97-fold, 3.09-fold and 8.24-fold, respectively. A similar interaction may occur with darunavir/cobicistat. Emtricitabine and tenofovir alafenamide do not interact with this metabolic pathway. |  |
| Darunavir/cobicistat | Coadministration with darunavir/cobicistat has not been studied and is not recommended as it may substantially increase glecaprevir exposure. Medicinal products that inhibit OATP1B1/3 (e.g. darunavir) increase systemic concentrations of glecaprevir. Coadministration of darunavir/ritonavir (800/100 mg) increased glecaprevir AUC, Cmax and Cmin by 4.97-fold, 3.09-fold and 8.24-fold, respectively. A similar interaction may occur with darunavir/cobicistat. |  |
| Desogestrel/ethinylestradiol (COC) | Coadministration of glecaprevir/pibrentasvir and doses of >20 μg ethinylestradiol is contraindicated. In interaction studies evaluating ethinylestradiol and glecaprevir/pibrentasvir in healthy female subjects, ALT elevations were observed in some subjects during coadministration. Based on increased ALT levels, coadministration of ethinylestradiol-containing hormonal contraceptives or hormone replacement therapies is not recommended. |  |
| Drospirenone/ethinyl estradiol (COC) | Coadministration with glecaprevir/pibrentasvir and doses of >20 μg ethinylestradiol is contraindicated. In interaction studies evaluating ethinylestradiol and glecaprevir/pibrentasvir in healthy female subjects, ALT elevations were observed in some subjects during coadministration. Based on increased ALT levels, coadministration of ethinylestradiol-containing hormonal contraceptives or hormone replacement therapies is not recommended. |  |
| Efavirenz | Coadministration is not recommended as efavirenz may reduce glecaprevir/pibrentasvir concentrations and this may lead to reduced therapeutic effect. Coadministration of glecaprevir/pibrentasvir and efavirenz/emtricitabine/tenofovir-DF (600/200/300 mg once daily) was studied to determine the effect on tenofovir concentrations. The effect on glecaprevir and pibrentasvir was not directly quantified within this study, but glecaprevir and pibrentasvir exposures were significantly lower than historical controls. |  |
| Eltrombopag | Coadministration is contraindicated. Concomitant use of glecaprevir/pibrentasvir with OATP1B inhibitors, such as eltrombopag, may increase the risk of ALT elevations due to a significant increase in pibrentasvir plasma concentrations caused by OATP1B1/3 inhibition. |  |
| Ethinyl estradiol | Coadministration with doses of ethinylestradiol >20 μg is contraindicated. In interaction studies evaluating ethinylestradiol and glecaprevir/pibrentasvir in healthy female subjects, ALT elevations were observed in some subjects during coadministration. Based on increased ALT levels, coadministration of ethinylestradiol-containing hormonal contraceptives or hormone replacement therapies is not recommended. |  |
| Etravirine | Co-administration has not been studied but is not recommended. Concentration of glecaprevir/pibrentasvir may decrease due to CYP3A4 induction by etravirine which may lead to reduced therapeutic effect. |  |
| Fosamprenavir | Coadministration has not been studied and is not recommended. Concomitant use of glecaprevir/pibrentasvir with OATP1B inhibitors, such as fosamprenavir, may increase the risk of ALT elevations due to a significant increase in glecaprevir/pibrentasvir plasma concentrations caused by OATP1B1/3 inhibition. Based on interactions studies, co-administration of glecaprevir/pibrentasvir with ritonavir-boosted HIV protease inhibitors is not recommended. |  |
| Ledipasvir/Sofosbuvir | Coadministration of glecaprevir/pibrentasvir with other directly acting antivirals has not been studied and therefore cannot be recommended. |  |
| Levonorgestrel/ethinyl estradiol (COC) | Coadministration with glecaprevir/pibrentasvir and doses of >20 μg ethinylestradiol is contraindicated. In interaction studies evaluating ethinylestradiol and glecaprevir/pibrentasvir in healthy female subjects, ALT elevations were observed in some subjects during coadministration. Based on increased ALT levels, coadministration of ethinylestradiol-containing hormonal contraceptives or hormone replacement therapies is not recommended. Coadministration of ethinylestradiol/levonorgestrel (20/100 µg once daily) and glecaprevir/pibrentasvir (300/120 mg once daily) increased ethinylestradiol Cmax, AUC and Cmin by 30%, 40% and 56%, respectively; Cmax, AUC and Cmin of norgestrel increased by 37%, 68% and 77%, respectively (n=12). No effect on glecaprevir/pibrentasvir was observed. |  |
| Lopinavir | Coadministration is not recommended. Concomitant use of glecaprevir/pibrentasvir with OATP1B inhibitors, such as lopinavir, may increase the risk of ALT elevations due to a significant increase in glecaprevir/pibrentasvir plasma concentrations caused by OATP1B1/3 inhibition. Coadministration of lopinavir/ritonavir and glecaprevir/pibrentasvir increased glecaprevir Cmax, AUC and Cmin by 2.55-fold, 4.38-fold and 18.6-fold; pibrentasvir Cmax, AUC and Cmin increased by 1.40-fold, 2.46-fold and 5.24-fold, respectively. |  |
| Nevirapine | Coadministration has not been studied and is not recommended. Coadministration may decrease glecaprevir/pibrentasvir concentrations due to induction of CYP3A4 by nevirapine which may lead to reduced therapeutic effect. |  |
| Nirmatrelvir/ritonavir | Coadministration is not recommended. Concomitant use of glecaprevir/pibrentasvir with OATP1B inhibitors, such as ritonavir, may substantially increase glecaprevir/pibrentasvir plasma concentrations and thereby increase the risk of ALT elevations. Consider pausing glecaprevir/pibrentasvir during and up to 3 days after the completion of nirmatrelvir/ritonavir treatment. However, the risk/benefit of prescribing nirmatrelvir/ritonavir should be carefully evaluated in patients with suboptimal adherence to glecaprevir/pibrentasvir as further treatment interruption could potentially lead to HCV treatment failure. |  |
| Norethisterone (Norethindrone)/ethinyl estradiol (COC) | Coadministration with glecaprevir/pibrentasvir and ethinylestradiol is contraindicated. In interaction studies evaluating ethinylestradiol and glecaprevir/pibrentasvir in healthy female subjects, ALT elevations were observed in some subjects during coadministration. Based on increased ALT levels, coadministration of ethinylestradiol containing hormonal contraceptives or hormone replacement therapies is not recommended. |  |
| Norgestrel/ethinyl estradiol (COC) | Coadministration with glecaprevir/pibrentasvir and ethinylestradiol is contraindicated. In interaction studies evaluating ethinylestradiol and glecaprevir/pibrentasvir in healthy female subjects, ALT elevations were observed in some subjects during coadministration. Based on increased ALT levels, coadministration of ethinylestradiol containing hormonal contraceptives or hormone replacement therapies is not recommended |  |
| Phenobarbital | Coadministration has not been formally studied and is not currently recommended. Induction of CYP3A4 and/or P-gp by phenobarbital may significantly decrease plasma concentrations of glecaprevir/pibrentasvir; this may lead to reduced therapeutic effect and potential virological failure. Note - there are a small number of cases reported of patients who have remained on an inducing antiepileptic during HCV DAA therapy (including with glecaprevir/pibrentasvir) and achieved a sustained virologic response. Although every effort to prevent concomitant use of glecaprevir/pibrentasvir and potent inducers should be made, these cases have demonstrated that clinical cure may still be achieved in patients where coadministration cannot be avoided. Therapeutic drug level monitoring could be considered, where available. |  |
| Phenytoin | Coadministration has not been formally studied and is not currently recommended. Induction of CYP3A4 and/or P-gp by phenytoin may significantly decrease plasma concentrations of glecaprevir/pibrentasvir; this may lead to reduced therapeutic effect and potential virological failure. Note - there are a small number of cases reported of patients who have remained on an inducing antiepileptic during HCV DAA therapy (including with glecaprevir/pibrentasvir) and achieved a sustained virologic response. Although every effort to prevent concomitant use of glecaprevir/pibrentasvir and potent inducers should be made, these cases have demonstrated that clinical cure may still be achieved in patients where coadministration cannot be avoided. Therapeutic drug level monitoring could be considered, where available. |  |
| Primidone | Coadministration has not been formally studied and is not currently recommended. Induction of P-gp by primidone may significantly decrease plasma concentrations of glecaprevir/pibrentasvir; this may lead to reduced therapeutic effect and potential virological failure. Note - there are a small number of cases reported of patients who have remained on an inducing antiepileptic during HCV DAA therapy (including with glecaprevir/pibrentasvir) and achieved a sustained virologic response. Although every effort to prevent concomitant use of glecaprevir/pibrentasvir and potent inducers should be made, these cases have demonstrated that clinical cure may still be achieved in patients where coadministration cannot be avoided. Therapeutic drug level monitoring could be considered, where available. |  |
| Rifabutin | Coadministration has not been studied and is not recommended. Induction of P-gp by rifabutin may significantly decrease plasma concentrations of glecaprevir/pibrentasvir leading to a reduced therapeutic effect. |  |
| Rifampicin | Coadministration is contraindicated. Concentrations of glecaprevir/pibrentasvir may be significantly decreased due to strong CYP3A induction after multiple doses of rifampicin. This may lead to loss of virologic response to glecaprevir/pibrentasvir. Coadministration of rifampicin (600 mg once daily) and glecaprevir/pibrentasvir decreased glecaprevir Cmax and AUC by 86% and 88%; pibrentasvir Cmax and AUC decreased by 83% and 87%. Note, following coadministration with a single dose of rifampicin, concentrations of glecaprevir were substantially increased by 8.55-fold, due to inhibition of OATP1B1/3. | ✓ |
| Ritonavir | Coadministration is not recommended. Concomitant use of glecaprevir/pibrentasvir with OATP1B inhibitors, such as ritonavir, may increase glecaprevir/pibrentasvir plasma concentrations. |  |
| Simvastatin | Coadministration is contraindicated due to the increased risk of myopathy/rhabdomyolysis. Coadministration of glecaprevir/pibrentasvir and simvastatin (5 mg once daily) increased simvastatin Cmax and AUC by 99% and 132%; Cmax and AUC of simvastatin acid increased by 10.2-fold and 4.48-fold. |  |
| Sofosbuvir/Velpatasvir | Coadministration of glecaprevir/pibrentasvir with other directly acting antivirals has not been studied and therefore cannot be recommended. |  |
| Vinblastine | Coadministration has not been studied and is not recommended. Vinblastine is a substrate of P-gp and concentrations may increase due to inhibition of P-gp by glecaprevir/pibrentasvir. This may lead to severe adverse effects. |  |
| Vincristine | Coadministration has not been studied and is not recommended. Vincristine is a substrate of P-gp and concentrations may increase due to inhibition of P-gp by glecaprevir/pibrentasvir. This may lead to severe adverse effects. |  |

JPI: Japanese package inserts
